# OpenStats: how to combine statistics and research data management (RDM) to leverage efficient scientific data analysis by guided statistics

**DOI:** 10.1186/s13321-026-01241-2

**Published:** 2026-07-01

**Authors:** Konrad Krämer, Pierre Tremouilhac, Fabian Mauz, Christoph Grathwol, Nicole Jung, Stefan Bräse

**Affiliations:** 1 Institute of Biological and Chemical Systems, Functional Molecular Systems (IBCS), Karlsruhe Institute of Technology, Kaiserstraße 12, 76131 Karlsruhe, Germany; 2 Karlsruhe Nano Micro Facility (KNMFi), Karlsruhe Institute of Technology, Kaiserstraße 12, 76131 Karlsruhe, Germany; 3 Institute of Organic Chemistry, Karlsruhe Institute of Technology, Kaiserstraße 12, 76131 Karlsruhe, Germany; 4 Department of Bioorganic Chemistry, Leibniz Institute of Plant Biochemistry (IPB), Weinberg 3, 06120 Halle (Saale), Germany

## Abstract

We developed OpenStats, a user-friendly web application that brings the power of the R language to researchers through a high-level interface and broad support for statistical methods such as t-tests and ANOVA. OpenStats was integrated into our electronic lab notebook Chemotion ELN via its third-party API, enabling direct data exchange with it. A pivotal feature of OpenStats is its ability to record each analysis step in a structured history. This record allows users to retrace their work and enables automatic replay of the entire analysis, promoting reproducibility and long-term data integrity. In the modern research landscape, Research Data Management (RDM) tools like electronic lab notebooks (ELNs) are crucial for generating reproducible, repeatable, and transparently documented data. However, integrating statistical analysis tools into RDM systems remained a challenge. We demonstrated how OpenStats can be seamlessly linked to Research Data Management (RDM) systems, using Chemotion ELN as a reference implementation, by our application to a standard dose–response assay scenario. This linkage allows the integration of statistical data analysis in the form of a closed, traceable workflow into the RDM world. It therefore complements systems that are aiming for reproducible and standardized workflows in the scientific environment.

**Supplementary Information:**

The online version contains supplementary material available at 10.1186/s13321-026-01241-2.

## Scientific contribution

By advancing the reproducibility and transparency of scientific data and the establishment of standardized routines to enable comparability of data we address a persistent challenge in scientific data evaluation and reporting. OpenStats brings statistical evaluation combined with analysis history tracking into the daily routine of scientists—even if they are not experts for statistics and not familiar with common statistics tools. The integration of OpenStats into RDM systems supports complete reproducibility by allowing automatic replay of the entire workflow enabling a closed, traceable, and standardized workflow from data acquisition to statistical evaluation.

**Supplementary Information:**

The online version contains supplementary material available at 10.1186/s13321-026-01241-2.

## Introduction

In modern scientific research, efficient, reproducible, and standardized workflows are essential for planning experiments, managing data, and analyzing results [1, 2]. Research Data Management (RDM) systems such as Electronic Lab Notebooks (ELNs) facilitate these processes by providing digital environments for experiment planning, data storage, data analysis, and documentation [3–5]. While discipline agnostic ELNs usually offer a broad applicability in different domains, but lack functions to deal with data structures and content established in the communities [6, 7], domain-specific systems offer diverse functions that support scientists in their work, but are usually limited in terms of suitability for other domains [8, 9]. Still, the domain-specific ELNs play a pivotal role in RDM as they allow for the storage of structured data that follows the standards and routines of the respective communities. In addition, they include the required functionality to parse data and validate data to ensure the correct assignment of results to the established data structure [10]. Besides the very broad functionality that can be found within the feature lists of ELNs that are currently on the market, ELNs used by academic scientists [11] lack solutions to systematically integrate statistics if it comes to requirements exceeding a general R, python or jupyter interface. This is even more problematic as statistical analysis should be a fundamental component of research, playing a crucial role in data exploration and hypothesis testing [12–15]. ELNs not only offer the possibility to integrate statistical methods into a virtual working environment for scientists in order to make work more seamless and tools more interconnected. They also provide the opportunity to systematically establish validated statistical routines and methods more broadly, thereby contributing to progress toward the standardization and reproducibility of statistical experiments by providing proven procedures. The availability of validated statistical elements in ELNs could offer particular added value, as fundamental routines can be established across communities, independent of the availability of specific tools within individual research groups and their particular workflows, which sometimes also vary from one scientist to another. The definition and implementation of such a statistics support that serves the needs of the scientists is quite challenging: The complexity and diversity of statistical methods are high, and they differ a lot depending on the required scientific question, and connected to that, the need to define and configure the available tools to allow a suitable application. For example, descriptive statistics facilitate the initial examination of datasets by computing characteristic values such as the mean, median, and standard error, as well as visualizing distributions. In contrast, inferential statistics enable researchers to identify significant differences between groups through various tests, including t-tests and ANOVA [16–18]. In addition, in statistical analysis, researchers often rely on a variety of statistical tools to process and interpret their data, and the choice of software significantly impacts efficiency, accessibility, and reproducibility. Concerning a potential integration of statistics into an ELN functionality, this means that an ELN would probably need to support different statistics tools and would need to reflect the needs of the scientists in terms of flexible and adaptable use. For example, Microsoft Excel [19] is widely used for statistical analysis, particularly in biological research [20, 21]. However, Microsoft Excel lacks built-in support for many advanced statistical methods. Performing an analysis of variance (ANOVA) requires the additional ToolPak extension, and further assumption testing necessitates external plugins such as the Real Statistics Resource Pack [22, 23]. To address these limitations, various proprietary statistical software solutions are available, including SPSS [24], Minitab [25], and GraphPad Prism [26]. While these tools provide more robust statistical capabilities, they are not universally accessible due to their cost, restricting use to those with financial resources. As a result, reliance on such software undermines the reproducibility of data analysis by limiting accessibility. Furthermore, there is no recorded workflow or analysis history available, making it impossible to repeat or verify the statistical analysis. An alternative approach involves the use of open-source programming languages such as R [27] and Python [28], which offer a vast array of statistical tests and ensure a fully reproducible and repeatable workflow. While the application of R and Python requires at least basic programming skills, there are free software tools such as Jamovi, JASP, and BlueSky Statistics that offer a user-friendly interface, meaning that no programming skills are required. However, these tools cannot be seamlessly integrated into a workflow with web-based services such as Chemotion ELN, as they either do not support a browser version (BlueSky Statistics) or only provide a proprietary web version (Jamoni). Moreover, the replay of an analysis in these tools is session-based.

## Results and discussion

### General considerations

The work described here aims to provide an R-based statistical tool that covers basic routines for statistical analysis. The tool should be available as a stand-alone solution and should be integrated into a research data environment to demonstrate the benefits of including statistics directly into the workflow of experiment conduction and analysis in a research data management tool. We aim to demonstrate how statistics can be easily provided to ELN users, regardless of their programming skills or familiarity with using Jupyter notebooks with an ELN, or configuring APIs. Furthermore, we demonstrate that our solution allows for establishing standardized ways for statistical analysis with open source tools, enabling the harmonization of methods and results in a community that currently applies multiple different tools and methods, mostly only accessible to individual scientists or within a working group. In this way, our work should allow the introduction of standard routines to foster comparability of statistical analysis.

To enhance the reproducibility and transparency of experiments and their analysis, methods should be established within the developed software to record the conducted steps. This ensures full traceability of all performed actions and enables the experimental analysis to be repeated under identical conditions.

Our approach resulted in the development of OpenStats, a statistical software that allows us to analyze assays conducted by biochemists, chemical biologists, and biologists. Moreover, we established a seamless communication between OpenStats and Chemotion ELN, chosen as the reference implementation for its alignment with our application domain in chemistry and biology, and its support for well-structured data compliant with domain-specific standards.

### Development of the statistics application

The statistics application OpenStats was developed as a web-based platform with a user interface (UI) that seamlessly integrates R-based analyses into the workflow of scientists. While it can be used as a standalone application, it was specifically designed to integrate efficiently as a third-party app (TPA) within the electronic lab notebook (ELN). The programming language R [29] enables scientists to run statistical analyses of their data at a high level of professionalism. The application uses the package shiny [30, 31], released in 2012, enabling the creation of interactive web applications directly from R.

OpenStats offers many statistical tests provided by R itself, for instance, the t-test, correlation tests, and the ANOVA [32]. Moreover, for linear models, post hoc tests from the agricolae [33] package are offered, whereas for generalised linear models, the emmeans package [34] is used. Furthermore, for the visualization of data, OpenStats employs the R package ggplot2 [35], which has become a standard for data visualization since its release in 2007. In the following sections, many capabilities, including the dose–response analysis, which uses the drc package [36], are presented in detail. In addition, OpenStats offers many other useful tools required by biologists and chemists. The creation of summary statistics, calibration curves, and parameter fitting are presented in the supplementary Section 7. Mentionable is that the dependencies required for the statistical tests are R packages which have shown stable and well-maintained interfaces over many years, making them suitable for reproducible workflows.

A key feature of OpenStats is its ability to execute text-based R commands, for instance, for Data wrangling actions. To ensure security and server-side execution, OpenStats processes each expression using its Abstract Syntax Tree (AST) representation. The system then applies the following validation steps: (1) Function Permission Check ensuring that only permitted functions are executed, (2) Variable Existence Check, verifying that all referenced variables exist within the dataset, (3) Encapsulated Evaluation ensuring that all variables are placed in a dedicated R environment, isolating the execution and preventing unintended modifications. These validation measures ensure that users can only execute specific, pre-approved functions, effectively preventing the execution of potentially malicious code. This is particularly important because R provides functions that could otherwise be used to access the underlying system. By enforcing these restrictions, OpenStats mitigates security risks while maintaining a controlled and safe execution environment.

### ELN integration

Chemotion ELN supports different ways to work with data that requires discipline-specific tools (see supplementary Sect.  1). For instance, software can be embedded into the UI and/or backend of the ELN, being then deeply integrated into the ELN’s workflows. Alternatively, external software or services can be connected via application programming interfaces (APIs), thereby extending functionality without the need to install externally developed software within the system. A third option is to investigate analytical data from the ELN using proprietary software installed locally. Once the analysis is complete, the results are automatically saved back to the ELN. For the use of OpenStats, we decided on another implementation that allows seamless communication between Chemotion ELN and third-party applications (TPAs) running on a server and can be used either via the ELN or independently of it. This approach has several advantages over the other solutions: (1) the integration of a TPA into the ELN workflow makes the main application independent of external software dependencies and fosters software modularity. (2) The connection of a TPA allows the ELN admin to allow as many TPAs as requested from the ELN users without the risk of overly complex ELN architectures. (3) The third-party app allows the integration of user input during the analysis process and is, therefore, more flexible than the connection of external services via API. (4) The development of a TPA allows a compromise between flexibility and standardization. Different use cases can be addressed by providing dedicated TPAs, and once a TPA is developed for a specific purpose, the entire community can benefit from its availability through the ELN. The TPA could easily introduce standards that are currently not established in the community.

### Data processing workflow and standalone use

**Fig. 1:**
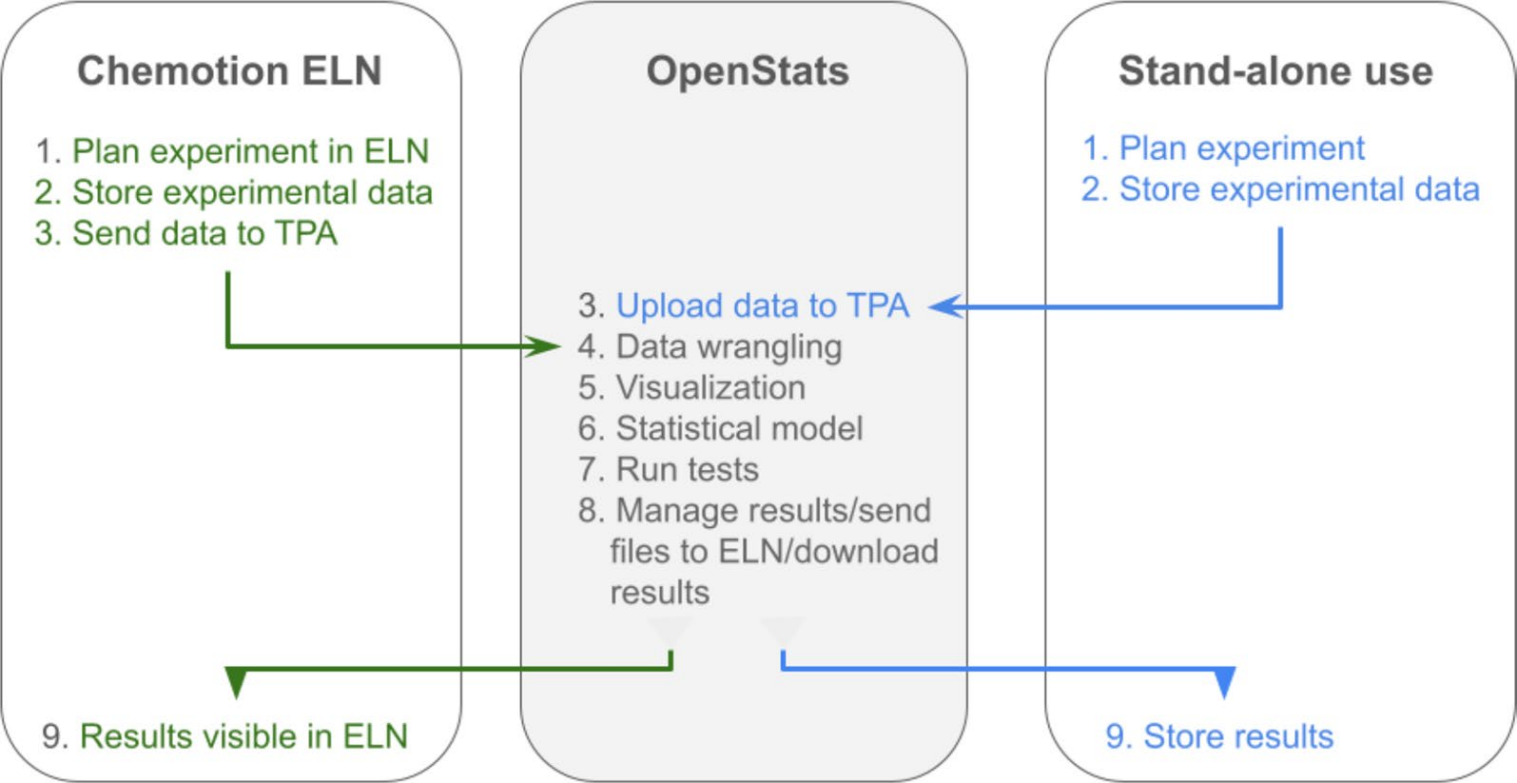
Schematic representation of the data analysis workflow integrating Chemotion ELN with OpenStats as a TPA compared to using OpenStats as a stand-alone version. For the ELN-integrated use of OpenStats, experiments are planned and stored within Chemotion ELN before relevant data files are transferred to the TPA. The processed results and the history are then returned to Chemotion ELN. If used as stand-alone software, the planning and storage of data need to be organized by the user, and the TPA analysis is started by uploading data to the TPA directly

OpenStats can be used as a stand-alone software or integrated into Chemotion ELN. The two options are compared in Fig. 1, showing the differences in the research management workflow and the steps that are common. Due to the manifold benefits of using OpenStats as integrated software, details of the workflow are described for the scenario that includes Chemotion ELN in the following sections. Chemotion ELN and OpenStats interact in a way that allows efficient, semi-automated, and reproducible statistical analysis in the form of a pipeline (Fig. 1). Chemotion ELN supports experiment planning (1), structured data storage (2), and the seamless transfer of data to web applications (3). OpenStats then enables data wrangling (4), data visualization (5), statistical model creation and application (6), conducting statistical tests (7), and sending the results back to the ELN (8), where the results can be accessed (9).

### Step 1: experiment planning

**Fig. 2:**
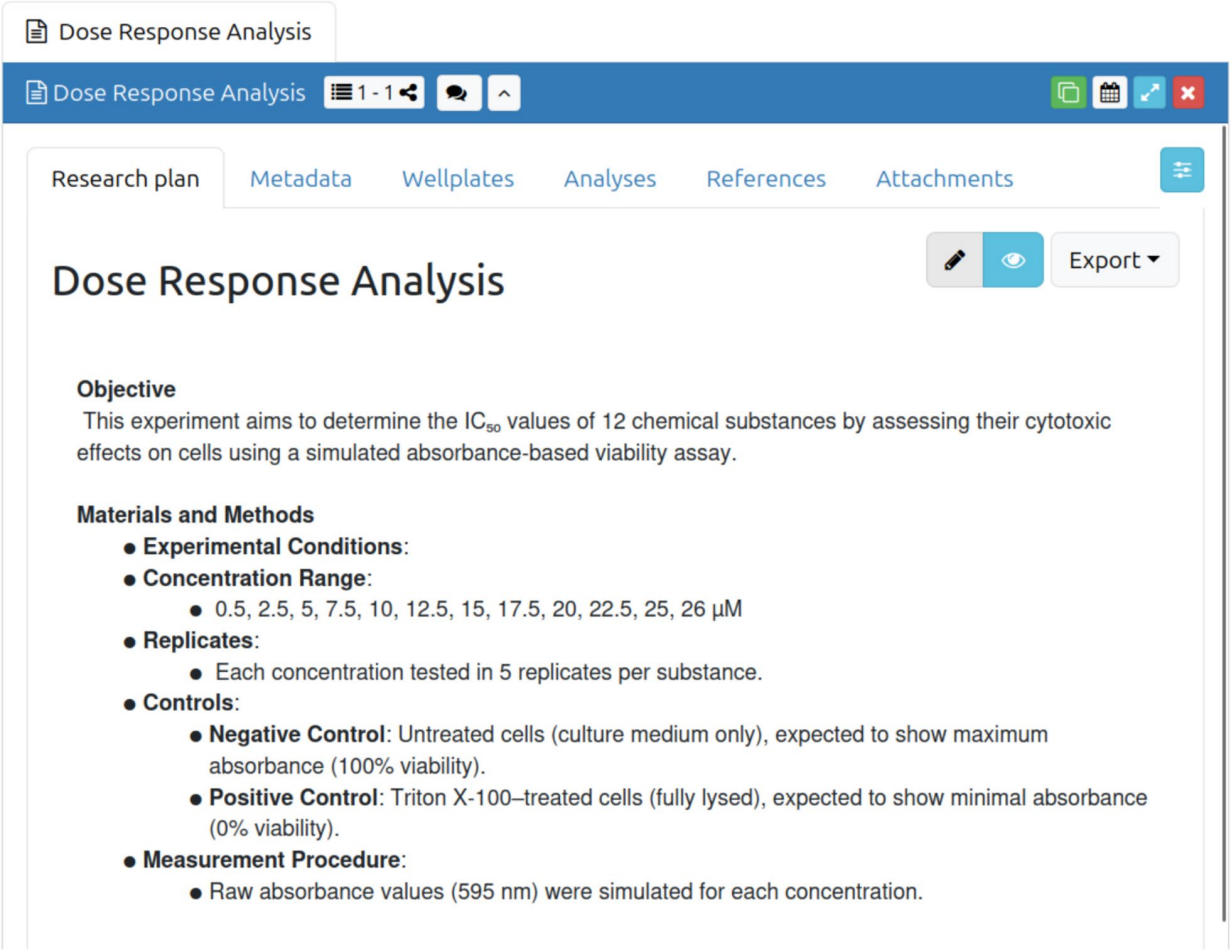
Experiment planning in Chemotion ELN. The ELN content area *Research plan* documents the objective and experimental setup for a dose–response assay aimed at determining IC_50_ values of 12 chemical substances across 12 concentration levels. It includes concentration ranges, replicates, controls, and measurement procedures based on simulated absorbance values

To demonstrate the experiment planning process, we simulated a dose–response dataset, mimicking a common colorimetric assay (MTT (3-(4,5-dimethylthiazol-2-yl)-2,5-diphenyltetrazolium bromide) assay) for assessing metabolic activity, in the domain of chemistry, biochemistry, and biology. The exemplarily chosen setup comprises response trajectories for 12 distinct chemical substances, each measured in 5 replicates across 12 concentrations ranging from 0.5 to 26 µM (Fig. 2, for details of the design of the experiment see supplementary Section 2).

### Step 2 and step 3: storing results and sending them to TPAs

**Fig. 3:**
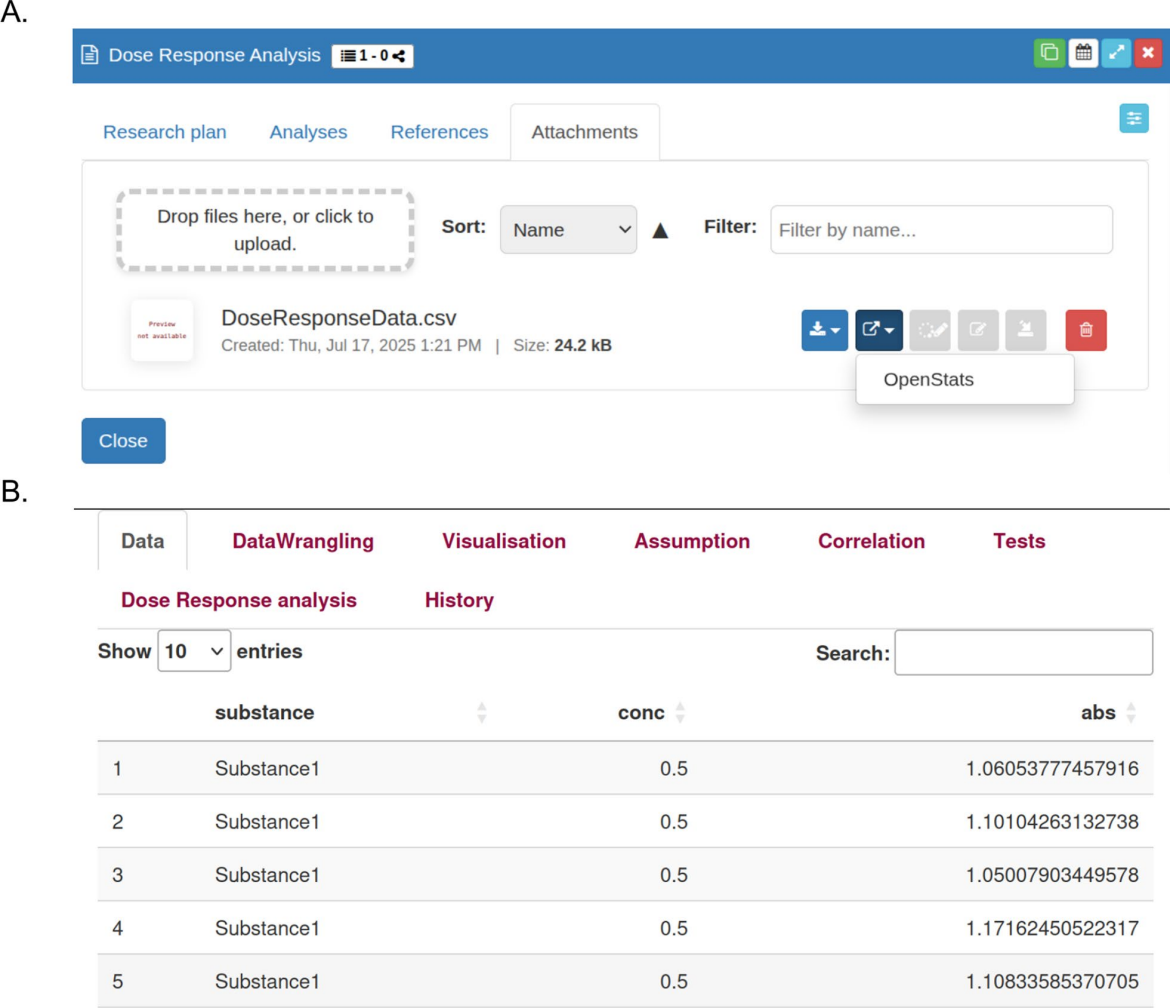
Transferring data to a third-party application (TPA) from Chemotion ELN. **A** Files are stored as attachments within Chemotion ELN. Selecting a TPA from the dropdown menu opens the application in a new browser tab. **B** Display the uploaded file's content in the OpenStats application, where the dataset is processed for statistical analysis

Once the measurements are completed, the data is uploaded to Chemotion ELN to the *Attachments tab* of the Research Plan (Fig. 3A) facilitated through a drop-down menu, which lists all available TPAs based on the file extension of the uploaded results file. By selecting a desired TPA, a new browser tab is automatically opened, transferring the file seamlessly to the selected service. Figure 3B illustrates this process, showing how the use of the OpenStats app is initiated by the app management, and how the DoseResponseData.csv file, successfully retrieved from Chemotion ELN, is displayed as a table for further analysis (see supplementary Section 3 for details).

### Step 4: data wrangling

**Fig. 4:**
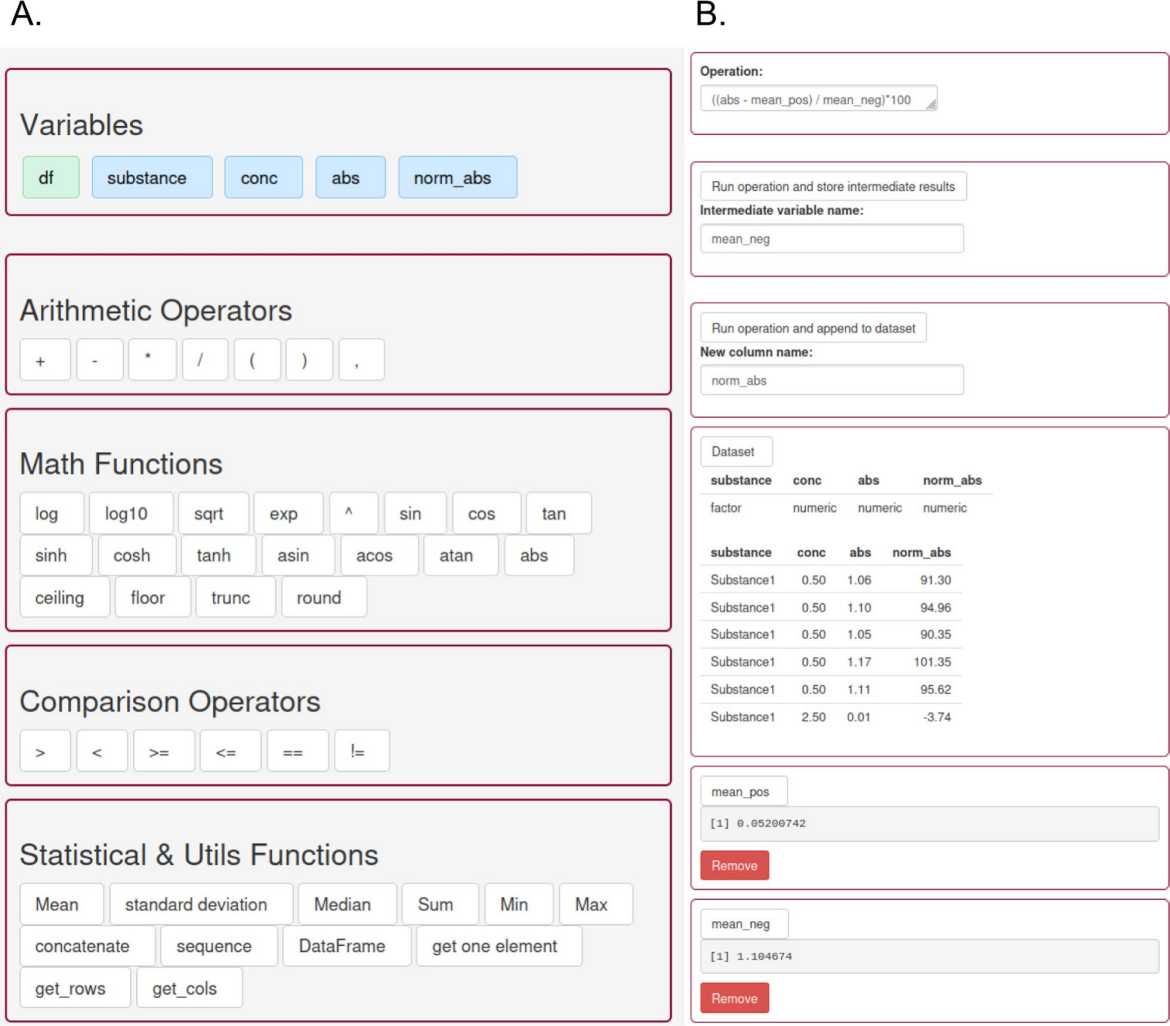
Data wrangling in OpenStats. **A** Subset of the left-side panel in the *Data Wrangling* tab. The “variables box” provides buttons referencing the entire dataset (df) and individual columns (substance, conc, abs, norm_abs). Additionally, arithmetic operations, mathematical functions, logical comparisons, and statistical functions are available for selection. **B** Main panel of the *Data Wrangling* tab. The upper section contains a text field where operations can be defined using the side panel buttons or manual input. Results can be stored as intermediate variables or appended to the dataset, with new column names specified in the designated input fields. The image illustrates an example where the *abs* column is normalized (*norm_abs*) using the means of the positive and negative controls, which were calculated beforehand

After loading the file into OpenStats, users can preprocess and manipulate their data within the *Data Wrangling* tab. A wide range of methods is available, enabling users to transform and analyze their dataset efficiently. The system is designed to be extensible, allowing the incorporation of additional data manipulation methods. To maintain a clear user interface, new functions can be structured as subtabs within the Data Wrangling section. To provide a guided and feasible application of functions to the data, the entire data wrangling process can be performed via predefined actions, eliminating the need for manual coding. Examples are given in Fig. 4: The first red box (Fig. 4 left) contains buttons that reference either the entire dataset named “df” (green) in the given example or individual columns (blue, names of the columns extracted from the dataset). Based on the selection, users can access a comprehensive set of operator buttons, including arithmetic, mathematical, logical comparison, and statistical functions, thereby applying them to the selected dataset or columns. When an operation is selected, it’s automatically added to the *Operations* text box in the main panel. For example, the expression “((abs—mean_pos)/mean_neg)*100” normalizes the absorbance values using the means of the positive and negative controls (Fig. 4). The mean values are computed beforehand and stored in custom-named intermediate variables (here: mean_pos and mean_neg). This normalization step transforms raw absorbance readings into percentage values relative to the defined control range. Advanced users can bypass the UI elements and enter text-based commands directly.

### Step 5: visualization

**Fig. 5:**
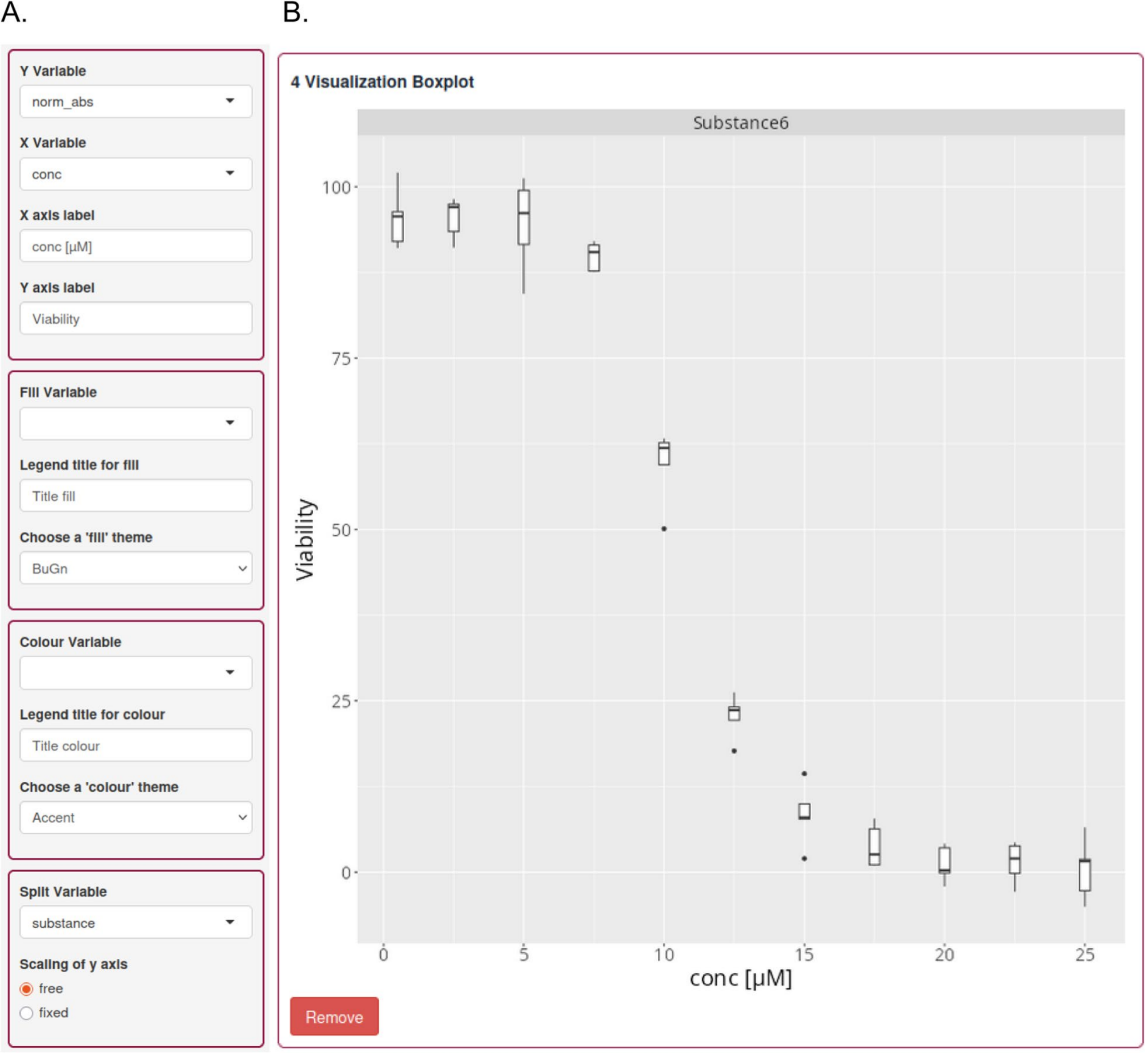
Data visualization in OpenStats. *Prior to* visualization, a filter was applied to display only the data for *Substance6*, ensuring clarity and consistency in the generated plots. **A** Sidebar excerpt showing the configuration options for plotting: selection of Y- and X-variables, customization of axis labels, grouping options using *fill* (for box interior) and *color* (for box borders), and the ability to create subplots using a *split variable*. **B** Resulting boxplot illustrating the relationship between viability and concentration for *Substance6*. Each box represents the distribution of normalized absorbance (viability) values across replicate measurements at each concentration

A crucial step in statistical data analysis is visualizing the dataset to identify patterns, trends, and relationships. To facilitate this, OpenStats provides boxplots, scatter plots, and line plots as plotting types. All visualizations are generated using ggplot2 [35, 37], a widely recognized R package known for its high-quality, customizable graphics. In Fig. 5 A, an excerpt of the available customization options is shown. To generate a plot, users must define the X- and Y-axis variables via a drop-down menu, which lists all dataset columns. The custom axis titles can be entered into the respective text fields.

Beyond the core settings shown in Fig. 5, users can incorporate information from additional columns in the dataset. Each group in the selected column is assigned a unique colour, applied to either the boxplot’s border or fill, making categorical differences visually distinct alongside the X and Y axes. Furthermore, plots can be split into multiple subplots -one for each group- allowing for clearer comparison across categories.

These features ensure that OpenStats provides a flexible and intuitive approach to data visualization, enhancing the interpretability of statistical results.

### Step 6: statistical models

**Fig. 6:**
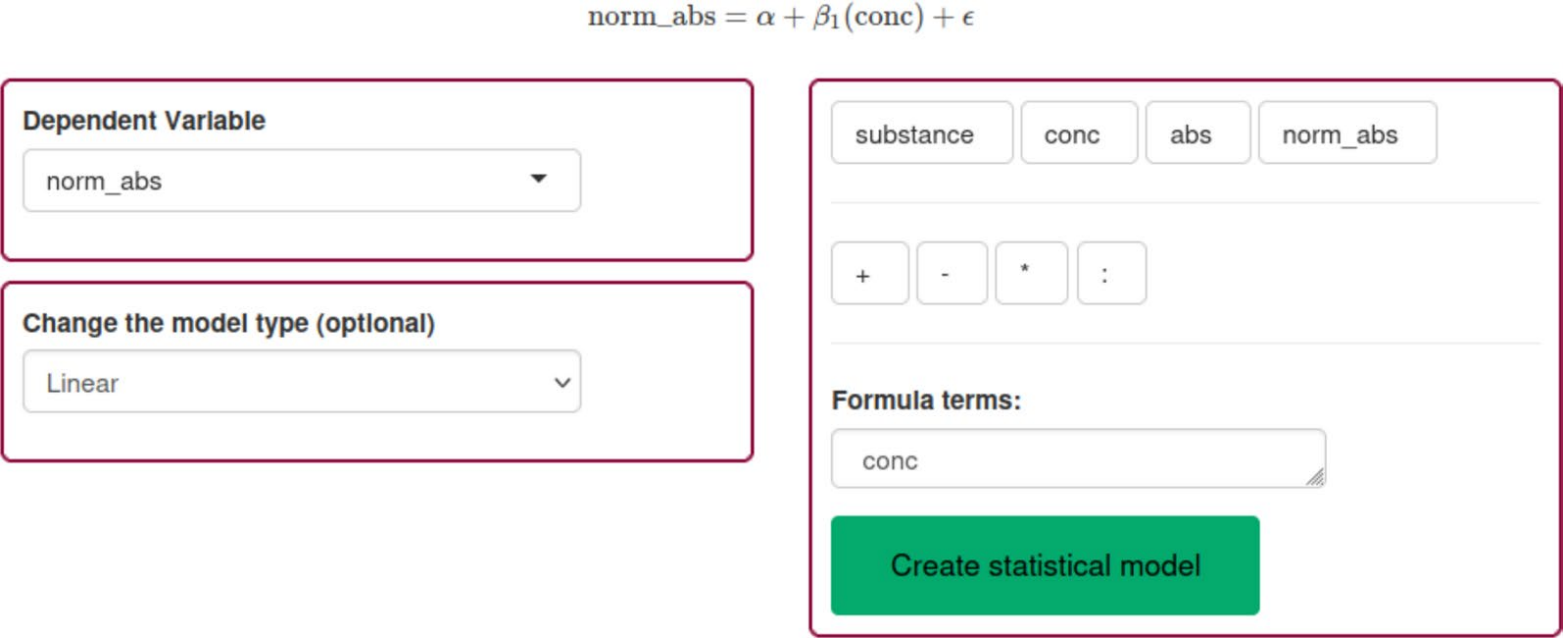
Constructing a statistical model in OpenStats. The *Formula Editor* guides users through defining a model by mapping variables. First, the normalized absorbance (*norm_abs*) is selected as the response (*dependent*) variable. Then, the concentration (*conc*) column is added as a predictor in the *Formula terms* field. The interface supports multiple predictors and interaction terms. In this example, conc is included as a main effect, but users can expand the model by adding more variables or specifying interactions

To conduct statistical tests in OpenStats, users must first define a statistical model. Currently, OpenStats supports linear models (lm), generalized linear models (glm), and a custom optimization model. Both linear models and generalized linear models are widely used and well-established methods for analyzing biological data [17]. In contrast, the optimization model requires users to define an explicit mathematical equation containing unknown parameters. This allows users to fit custom equations to experimental data—for example, to generate calibration curves. Although the optimization model is not a statistical model in the strict sense, it follows the same creation workflow as linear models and generalized linear models to maintain a consistent user experience. All models are created using the Formula Editor, which is accessible from all analysis tabs in OpenStats except the Data Wrangling tab, to avoid unnecessary interface clutter (Fig. 6). Within this window, users define the model by selecting a response variable (dependent variable) from a drop-down list of dataset columns (on the left), and adding predictor variables (independent variables) either via button clicks or by manually entering the formula in the text box. Notably, the set of available tests in the Assumptions and Tests tabs updates dynamically depending on the model type. For example, the t-test is available for linear models but not for generalized linear models. Moreover, some methods —such as correlation tests and dose–response analysis— do not rely on any of the statistical models (lm, glm, or optimization). However, for the sake of a streamlined and consistent interface, OpenStats still requires users to define a formula before running these analyses. In such cases, the formula serves only to map the response to predictor variables.

Figure 6 shows an example in which a linear model is created using normalized absorbance (*norm_abs*) as the response variable and concentration (*conc*) as the predictor. In the example, the formula is applied to the full dataset, which contains 12 different substances, and the resulting model pools all substances into a single fit. The corresponding model summary shows the estimated coefficients, standard errors, test statistics, and p-values, along with model selection metrics such as AIC (Akaike Information Criterion) and BIC (Bayesian Information Criterion). A fitted regression line with a 95% confidence band is also displayed (see supplement Section 4, Fig. S1). Although the pooling of heterogeneous substances into a single linear model is not statistically meaningful in itself, it provides a global visualization of the overall concentration–response pattern across the dataset. This artificial trend is not intended as a final model but instead serves as a first orientation step before applying substance-specific analyses.

### Step 7: Statistical tests

After creating the formula, the dose–response analysis is performed. Upon switching to the corresponding tab, the user must specify the column that contains the grouping variable—in this example, the column is named substance. Additionally, users may optionally apply a logarithmic scale to the x-axis, y-axis, or both by selecting the appropriate checkboxes. By clicking Conduct analysis, OpenStats fits individual dose–response models for each substance in the dataset using the specified formula.

**Fig. 7:**
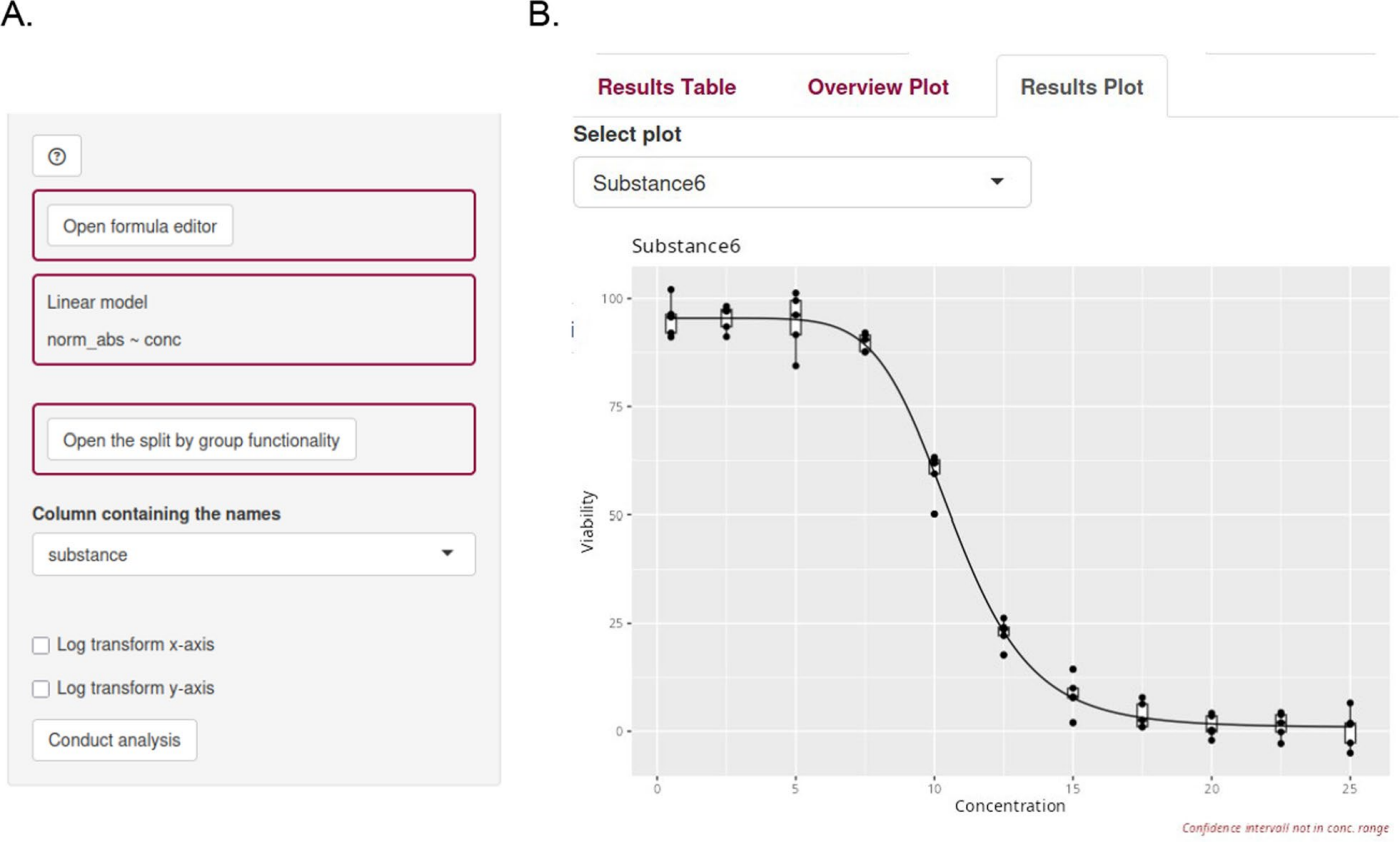
Dose–response analysis in OpenStats. **A** The left-hand sidebar shows the user interface elements required to run the analysis, including the selection of a previously defined model, specification of the grouping variable (substance), options for logarithmic scaling of the axes, and the button to initiate the dose–response analysis. **B** Plot of the raw viability values against concentration, overlaid with the predicted dose–response curve for *Substance6*

The results are presented in two forms. First, a summary table of all estimated parameters is provided (Fig. S2). The predicted values closely match the ground truth used to simulate the data. Second, the fitted models are visualized for each substance (Fig. 7). These plots show the raw viability values against concentration, overlaid with the predicted dose–response curve. This visualization allows users to assess model fit, identify deviations, and evaluate the quality of the underlying data. The app also includes an overview plot (not included in Fig. 7), which displays up to nine dose–response plots simultaneously to facilitate comparison across substances.

### Step 8: manage results and send files to ELN

**Fig. 8:**
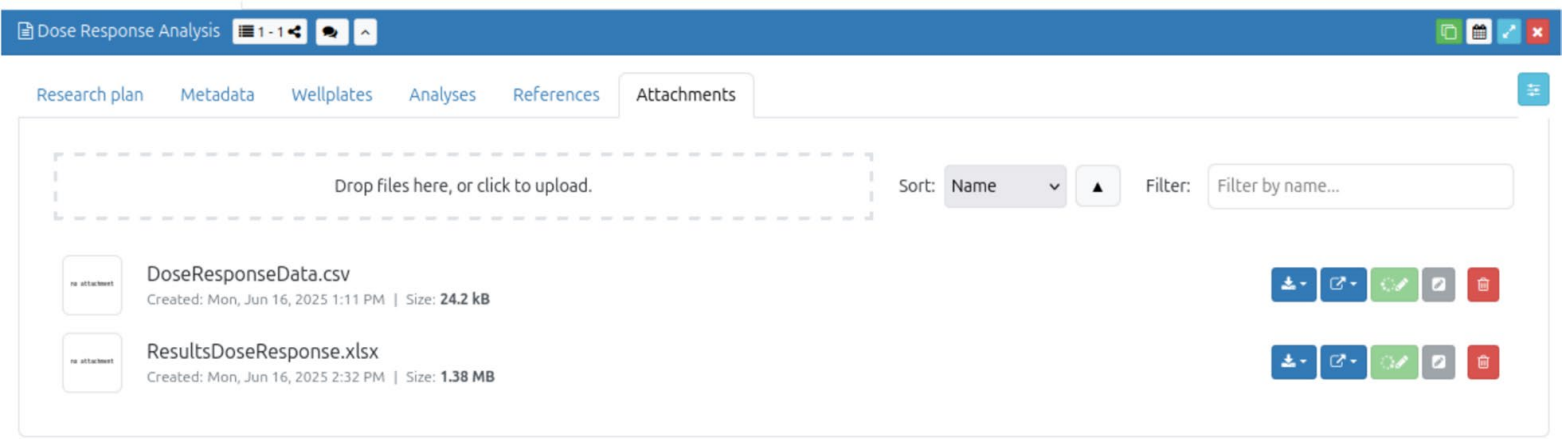
Returning statistical analysis results from OpenStats to Chemotion ELN. After completing the statistical analysis in OpenStats, the results are saved and transferred back to Chemotion ELN as a new file attachment. In this example, the analysis results are stored in ResultsDoseResponse.xlsx, which appears as a new attachment in the Research Plan, the same section where the original data file was initially sent to OpenStats

A key feature of OpenStats is the Result List, which provides an overview of all generated results across different tabs. Once satisfied with the results, the user must specify a file name before exporting the data (Fig. S1) and save the results to start the export process: when the app is running locally, the result list is downloaded as a ZIP archive, with each result saved as a separate file. In contrast, in the Chemotion ELN version, the entire result list is exported into a single Excel file and automatically transferred back to Chemotion ELN (Fig. 8), being stored in the same location within Chemotion ELN from which the original file was sent to OpenStats. This ensures a reproducible, closed-loop data analysis workflow, maintaining a seamless connection between data processing and experiment documentation.

### Reproducibility of analysis

A key strength of OpenStats is its ability to automatically record all analysis steps in a comprehensive history, stored alongside the actual results (Fig. S3). A history provided as a human-readable table allows users to trace the exact sequence of operations, including variable transformations, filters, visualizations, and statistical tests (Fig. S3A). This transparent documentation makes it easy to understand what was done and in what order, a feature that is often lacking in graphical user interfaces. In addition, the entire history is also stored in a machine-readable JSON format at the end of the resulting Excel file (Fig. S3B). This enables users to reload their dataset, enter the JSON history, and reproduce the complete analysis. This dual-format history system enables full reproducibility of statistical workflows without requiring users to write code. As such, it bridges the gap between interactive data analysis and the reproducibility typically only achievable through standardized environments or predefined workflows.

## Conclusion

OpenStats enables statistical analysis in R through an intuitive high-level user interface, offering an open-source alternative to proprietary tools in particular for chemists and biologists. It supports two main statistical models -linear and generalized linear- with a broad range of associated tests, such as ANOVA and post hoc comparisons. Notably, the test interface dynamically adapts to the selected model. In addition to these core models, users can perform nonlinear model fitting by defining a custom optimization model.

In addition, we established seamless communication between a research data management system (Chemotion ELN) and third-party applications, exemplified by OpenStats. Because both the Chemotion ELN and OpenStats are web-based, computationally intensive analyses can be offloaded to dedicated clusters. Through this integration, users can access these capabilities without handling data or files locally, enabling a closed, reproducible data analysis workflow directly within the ELN environment. Although it is also possible to use OpenStats locally as a standalone application.

Furthermore, a pivotal feature of OpenStats is its automatic recording of all user actions in a structured history, stored alongside the results, and enabling easy and efficient sharing between tools. This history not only allows users to retrace their analysis but also makes it possible to replay the entire workflow, enhancing transparency, reproducibility, and long-term traceability. To ensure compatibility with future updates, each history entry includes a version identifier, allowing workflows created in older versions to remain functional.

Thanks to this structured and forward-compatible architecture, OpenStats is well-positioned to evolve. While it already includes a wide range of statistical methods, its modular design allows for the straightforward addition of new models and tests.

## Supplementary Information

Supplementary Material 1.

## Data Availability

Project name: OpenStats. Project home page: https://github.com/ComPlat/OpenStats and Zenodo https://doi.org/10.5281/zenodo.14671306. Operating sytem(s): Platform independent. Programming language: R. Other requirements Chemotion ELN v.1.10.0 or higher (https://doi.org/10.5281/zenodo.10038678). License: GPL-3.0.

## Abbreviations

ELN: Electronic lab notebook
DB: Database
UI: User interface
API: Application programming interface
TPA: Third-party application
MTT: 3-(4,5-dimethylthiazol-2-yl)-2,5-diphenyltetrazolium bromide
LM: Linear model
GLM: Generalized linear model
