# Supplementary material for: OpenStats: how to combine statistics and research data management (RDM) to leverage efficient scientific data analysis by guided statistics
Source: J Cheminform. 2026 Jul 1;18:86. doi: 10.1186/s13321-026-01241-2 (PMC13330261; doi:10.1186/s13321-026-01241-2)
Supplement: Supplementary file 1 — Supplementary Material 1. [file 13321_2026_1241_MOESM1_ESM.pdf]

Supplemental Information

OpenStats: Using synergies of statistical tools and RDM methods to leverage the broad use of efficient data analysis tools by guided statistics

Konrad Krämer[a], Pierre Tremouilhac[a], Fabian Mauz[d], Christoph Grathwol[a], Nicole Jung[a,b], Stefan Bräse*[a,c]*

Email: nicole.jung@kit.edu; stefan.braese@kit.edu

Content

1. Chemotion ELN: Options to include software/services 2
2. Experimental details for dose response example 3
3. Data file requirements for import scenarios 4
4. The result list in OpenStats 4
5. Result of dose-response analysis 6
6. The history tab and its use to enhance reproducibility 7
7. Statistical applications in the field of Chemistry 8

1. Chemotion ELN: Options to include software/services

Chemotion ELN supports different ways to work with data that requires discipline-specific tools. For instance, software can be embedded into the UI and/or backend of the ELN, being then deeply integrated into the ELN's workflows. Alternatively, external software or services can be connected *via* application programming interfaces (APIs), thereby extending functionality without the need to install externally developed software within the system. A third option is to investigate analytical data from the ELN using proprietary software installed locally. Once the analysis is complete, the results are automatically saved back to the ELN. These options cover most standard user requirements but none is well suited for the integration of statistics tools due to various reasons: The integration of software and services via APIs are only possible if they are available as an open-source or under open access conditions, including the flexible adaptation for a certain scientific community. The embedding of larger software packages additionally suffers from a potentially complex ELN architecture and manifold dependencies derived from the additional packages. The last option, which relies on locally installed software, offers high flexibility but requires the individual scientist to install and properly configure the necessary statistical tools. To address these limitations, we present a method for enabling seamless communication between Chemotion ELN and third-party applications (TPAs) running on a server and can be used either via the ELN or independently of it. This approach has several advantages over the other solutions: (1) the integration of a TPA into the ELN workflow makes the main application independent of external software dependencies and fosters software modularity. (2) The connection of a TPA allows the ELN admin to allow as many TPAs as requested from the ELN users without the risk of overly complex ELN architectures.

(3) The third-party app allows the integration of user input during the analysis process and is, therefore, more flexible than the connection of external services via API.

(4) The development of a TPA allows a compromise between flexibility and standardization. For different applications, different TPAs can be provided, while once the TPA exists for a

certain application, the whole community may benefit from its availability through the ELN. The TPA could easily introduce standards that are currently not established in the community due to the use of different tools and methods.

2. Experimental details for dose response example

To demonstrate the experiment planning process, we simulated a dose–response dataset typical for common colorimetric assays (MTT assay) for assessing cell metabolic activity MTT in the domain of chemistry, biochemistry and biology. The exemplarily chosen setup comprisesing response trajectories for 12 distinct chemical substances, each substance measured in 5 replicates across 12 concentrations ranging from 0.5 to 26 µM (Fig. 2). The data points were generated using a four-parameter logistic (4PL) function (Eq. 1), with each substance assigned a different IC_{50} value: 1, 2.5, 6, 7.5, 9, 10.75, 12.5, 15.3, 17.5, 19.3, 22.2, and 24.5 µM. To simulate experimental variability, random noise was added to the computed response values.

$$Equation\ 1: lower\ limit\ +\ (upper\ limit\ -\ lower\ limit)\ /\ (1\ +\ (conc\ /\ IC50)^{slope})$$

Where:

- lower_limit: the minimum response level observed at high concentrations (e.g., complete inhibition)
- upper_limit: the maximum response level observed at low concentrations (e.g,. no inhibition)
- conc: the concentration of the compound
- IC50: the concentration at which the response is halfway between the lower and upper limits (i.e., 50% effect)

slope: the Hill slope, which determines the steepness of the curve

3. Data file requirements for import scenarios

Currently, *OpenStats* supports the import of excel, text and CSV files, common separators: “,”, “\t”, “ “ and “;” are automatically identified, facilitating seamless data integration. Multiple tables can be imported at once, provided they are separated by at least one empty row or column. Each table must have its column names defined in the first row.

4. The result list in OpenStats

Results are displayed in reverse chronological order, ensuring that the most recent output is always visible at the top (Supplementary Figure 3). This functionality allows users to quickly access and review previous analyses without navigating through multiple tabs. Additionally, if a result is no longer needed, it can be removed by clicking the red delete button located directly below the respective entry in the Result List. This streamlined approach enhances usability and workflow efficiency, ensuring that users can manage their statistical outputs effortlessly.

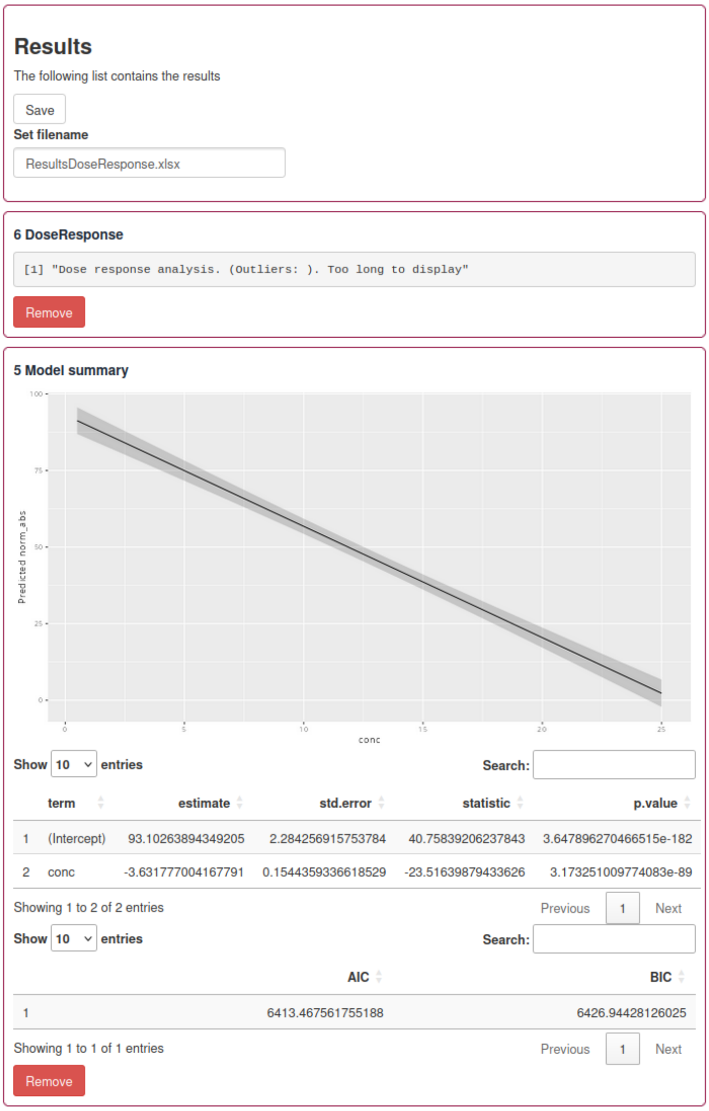

Supplementary Figure 1. The result list shows all results in reverse chronological order. The upper part of the *Result List,* which shows the last two results, the *6 DoseResponse,* is the result of the previously performed dose-response analysis. Due to its large size, the user can only see the results in the *Dose Response analysis* tab. Moreover, it is the result of the summary of the linear model displayed (*5 Model summary*). Additionally, results can be saved to a specified filename (e.g., ResultsDoseResponse.xlsx) and sent to Chemotion ELN.

5. Result of a dose-response analysis

A.

Results Table | Overview Plot | Results Plot

name	Response_lowestdose_predicted	Response_highestdose_predicted	HillCoefficient	asymptote_one	asymptote_two
Substance1	94.717700	0.231414	4.557450	0.231404	114.523206
Substance2	95.453100	0.249506	7.282070	0.249501	95.453866
Substance3	95.455900	-0.045942	6.701340	-0.052950	95.455862
Substance4	95.108200	0.115543	7.255540	0.099225	95.108182
Substance5	95.149400	0.474250	7.540470	0.430422	95.149383
Substance6	95.442500	1.054710	7.512920	0.896146	95.442524
Substance7	95.738300	1.445950	7.073360	0.794449	95.738284
Substance8	95.777200	3.178580	6.634780	-0.304894	95.777190
Substance9	95.623700	7.325020	6.578170	-1.355189	95.623715
Substance10	95.519200	13.462600	6.580320	-2.482638	95.519247
Substance11	95.436600	28.992800	6.501560	-7.441300	95.436593
Substance12	95.418000	44.344000	6.299750	-23.628628	95.418027

B.

IC50_relative	IC50_relative_lower	IC50_relative_higher	pIC50	RSE	p_value	Problems
0.704475	0.137817	1.271130	0.152134	3.896618	0.000000	
2.501480	2.444180	2.558770	-0.398197	3.896470	0.000000	
6.039270	5.873460	6.205090	-0.780984	3.866926	0.000000	
7.568760	7.406300	7.731220	-0.879025	3.864426	0.000000	
9.030820	8.844330	9.217310	-0.955727	3.833605	0.000000	
10.681100	10.479100	10.883000	-1.028620	3.829767	0.000000	
12.373400	12.130700	12.616100	-1.092490	3.855060	0.000000	
15.248400	14.897300	15.599500	-1.183220	3.855831	0.000000	
17.570900	17.018600	18.123100	-1.244790	3.869664	0.000000	
19.490200	18.558000	20.422500	-1.289820	3.880010	0.000000	
22.793100	19.988000	25.598300	-1.357800	3.886663	0.000000	
26.160400	17.289100	35.031800	-1.417640	3.887995	0.000000	IC50 larger than highest measured concentration

Supplementary Figure 2: Tabular output of a dose-response analysis in OpenStats. Each row corresponds to a specific substance in the dataset. The table summarizes key results from the model fitted by the drc package, including fitted values, confidence intervals, and test statistics. Due to representation issues, the table was split in part 2A. and 2B. (2B. continues the entries given in 2A.). Column explanations (left to right): name: The name or label of each tested group; Response_lowestdose_predicted: Model-predicted response at the lowest tested concentration; Response_highestdose_predicted: Model-predicted response at the highest tested concentration; HillCoefficient: the slope of the dose–response curve, indicating sensitivity to dose changes; asymptote_one / asymptote_two: The lower and upper

asymptotes of the fitted curve, corresponding to the minimum and maximum response levels; IC50_relative: The concentration at which 50% of the maximum effect is observed (inflection point of the curve); IC50_relative_lower / IC50_relative_higher: The lower and upper bounds of the 95% confidence interval for the IC50 estimate; pIC50: The negative base-10 logarithm of the IC50 value ($-\log_{10}$(IC50)), often used for improved interpretability and scale comparison; RSE: Residual standard error of the model fit, as reported by the drc summary; p_value: Significance of the model effect, calculated via noEffect(). Problems: A diagnostic message indicating model quality issues, such as insufficient response range or an IC50 estimate outside the concentration range.

6. The history tab and its use to enhance reproducibility

A.

HistoryTable		
Step	Result	details
Version		Nr: 1_2
CreateIntermediateVariable		operation: Mean(get_cols(get_rows(df, substance == "pos"), abs)); name: mean_pos
CreateIntermediateVariable		operation: Mean(get_cols(get_rows(df, substance == "neg"), abs)); name: mean_neg
CreateNewColumn		operation: ((abs - mean_pos) / mean_neg)*100; column name: norm_abs
ApplyFilter		Variable: substance; Variable levels: Substance6
Visualisation	4 Visualization Boxplot	x: conc; y: norm_abs; Plot-type: box; X axis label: conc [µM]; Y axis label: Viability; Type of x: factor; Colour variable: ; Legend title for colour: Title colour; Colour theme: Accent; Fill variable: ; Legend title for fill: Title fill; Fill theme: BuGn; Split in subplots: facet_wrap; Split by: substance; How to scale y in subplots: free; X-Range: c("0.25", "31.25"); Y-Range: c("-4.7840148304601", "107.194048477641"); Width: 10; Height: 10; Resolution: 300
RemoveFilter		Variable: ; Variable levels:
CreateFormula		formula: norm_abs ~ conc; Model Type: Linear; details:
ModelSummary	5 Model summary	formula: norm_abs ~ conc
DoseResponse	6 DoseResponse	Column containing the names: substance; Log transform x-axis: FALSE; Log transform y-axis: FALSE; formula: norm_abs ~ conc; outliers:

B.

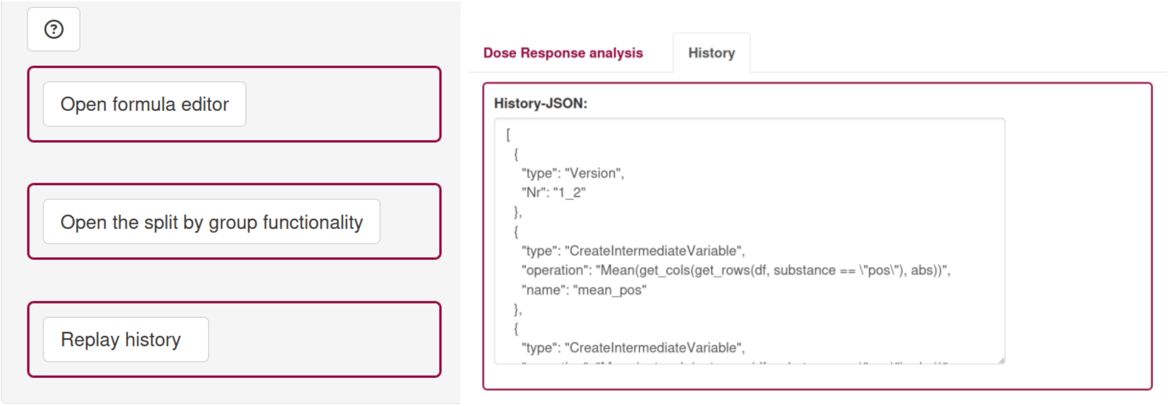

Supplementary Figure 3. Reproducibility *via* the history feature in OpenStats. 3A. At the beginning of the result file, the History Table provides a structured overview of all steps performed during the analysis, including variable creation, filtering, visualisation, and statistical modelling. Each step is listed chronologically with details on the operation performed, parameters used, and resulting outputs. 3B. At the end of the result file, the full analysis history

is stored in machine-readable JSON format. Users can reload the dataset, navigate to the History tab, paste the JSON content into the input field, and click Replay history to automatically reproduce all analysis steps. This feature ensures full reproducibility and facilitates collaborative workflows.

7. Statistical applications in the field of chemistry

OpenStats provides a broad range of statistical functionality tailored to the needs of biologists and chemists working with experimental data. In the following section, we describe in detail the procedures for summarizing data and constructing calibration curves.

A common experimental design in biology and chemistry involves comparing a response variable across multiple groups, for example, assessing the performance of different catalysts, as shown in the example (Fig. S4, data taken from an exemplary master thesis, KIT Karlsruhe). In many cases, more than one grouping factor is of interest. In OpenStats, such analyses can be performed in the Data Wrangling tab by selecting one or more grouping variables and subsequently applying a user-defined operation, the result of which is stored as a new variable. The specified expression is then automatically evaluated for each subset defined by the unique combinations of the selected grouping variables (Fig. S4).

A.

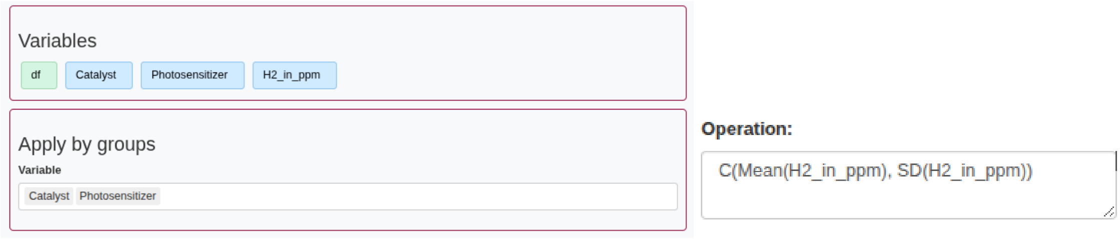

B.

name	value1	value2
Ni-Pyrrol.Ir(ppy)3	8876.33011764706	9206.641090164487
Ni-Pyrrol.Ru(bpy)3	5995.623324324324	7498.774640046636
Ni-Pyrrol.4CzIPN	1618.624	1414.715510712843
Ni-Pyrrol.Cu1	653.6831428571429	1079.457743337036
Ni-Pyrrol.Purpurin	0	0
Ni-Indol.Ru(bpy)3	3945.544333333333	5941.451431320405
Ni-Indol.Ir(ppy)3	15250.98876470588	30253.14049808037
Ni-Indol.4CzIPN	1416.767	1580.613479651945
Ni-Indol.Cu1	4186.908375	4476.421825098861
Ni-Indol.PP	0	0

Supplementary Figure 4. Calculation of summary statistics across groups. 4A**.** In the Data Wrangling tab, one or more grouping variables can be selected via the corresponding field in the sidebar. When an operation is specified and assigned to an intermediate variable, the expression (right panel) is evaluated separately for each data subset. These subsets are defined by the unique combinations (interactions) of the selected grouping variables. 4B. The resulting table reports the computed statistics for each group. The name column lists the corresponding combinations of the grouping variables, while the subsequent column(s) contain the results of the specified operation. In this example, the first column represents the mean, followed by the standard deviation.

Moreover, the quantification of substances typically relies on calibration curves, which can be constructed in OpenStats using the Formula Editor (Fig. S5). The example in Figure S5 shows simulated data based on a one-site binding equation, with additional Gaussian noise added. First, an appropriate model type is selected (Optimization Model). Subsequently, either a predefined functional form (such as standard binding or saturation models) can be chosen, or a custom equation can be specified using the free formula interface. Known variables (e.g., concentration and response) are mapped to the data, while unknown parameters are defined and estimated.

Parameter estimation is performed via numerical optimization, where the algorithm iteratively identifies the set of parameters that best fit the observed data according to a specified objective function. Optional settings, such as parameter bounds and random seeds, allow control over the optimization process and ensure reproducibility. This flexible framework enables the construction of simple linear calibration curves as well as the application of more complex, non-linear models. For example, it allows the estimation of kinetic parameters such as K_m in enzymatic assays or dissociation constants (e.g., K_d) in binding studies, thereby supporting a wide range of quantitative analyses in biological and chemical research.

A.

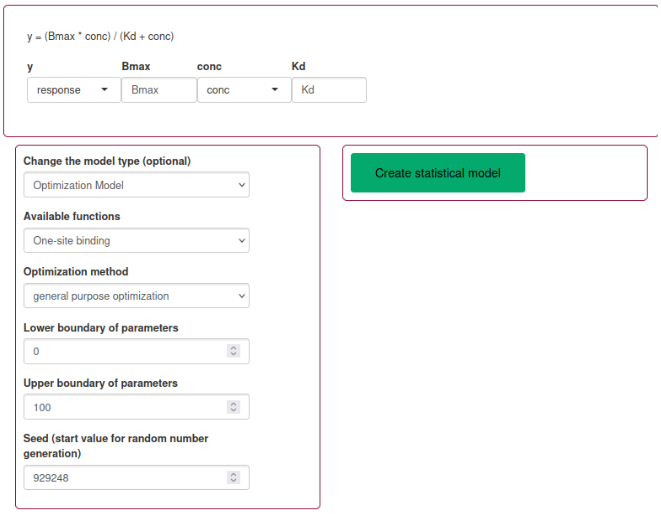

B.

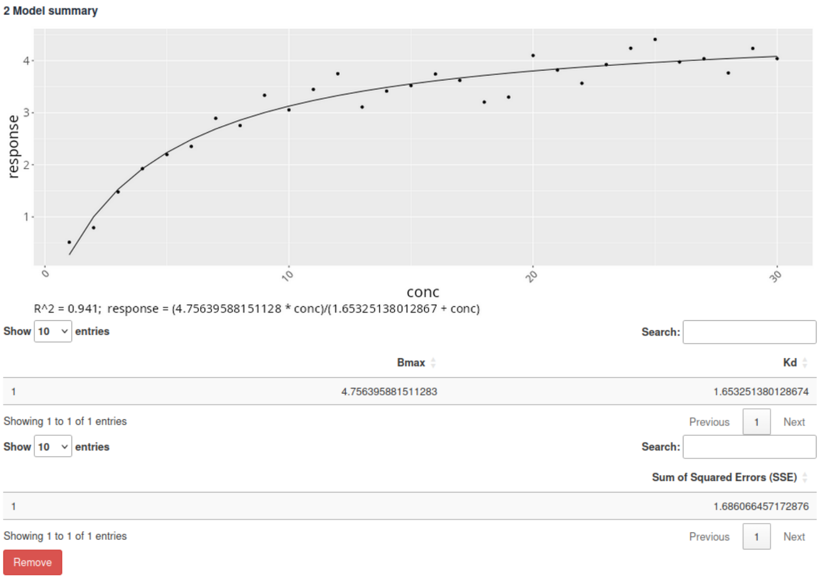

Supplementary Figure 5. Construction of calibration curves using optimization models. The data is simulated based on a one-site binding equation, with additional Gaussian noise added. 5A. In addition to linear and generalized linear models, the *Formula Editor* enables the specification of Optimization Models defined by user-selectable mathematical equations with known and unknown parameters. Predefined model forms (e.g., one-site binding, shown here) are available, and custom equations can be specified using *Free formula* in the available functions drop-down. In the example, *response* and *conc* represent the observed variables, while *Bmax* and *Kd* denote unknown parameters to be estimated (with optional renaming). The sidebar provides additional configuration options, including the choice of optimization algorithm, parameter bounds (used to determine suitable starting values), and a random seed to ensure reproducibility. 5B. Model summary of the fitted optimization model. Observed data

are displayed as points, and the fitted function is overlaid as a continuous line. The goodness-of-fit (R^2), estimated parameter values, and the sum of squared errors (SSE) are reported.
